# Supplementary material for: Transcranial focused ultrasound stimulation enhances semantic memory by modulating brain morphology, neurochemistry and neural dynamics
Source: Nat Commun. 2026 Feb 16;17:2833. doi: 10.1038/s41467-026-69579-7 (PMC13022233; doi:10.1038/s41467-026-69579-7)
Supplement: Supplementary file 1 — Supplementary Information [file 41467_2026_69579_MOESM1_ESM.pdf]

# Supplementary information

Behavioural results

Supplementary Figure 1

Supplementary Figure 2

Supplementary Table 1

Supplementary Table 2

Supplementary Table 3

Supplementary Table 4

Behavioural results

A 2 x 2 repeated measures ANOVA was conducted with stimulation (ATL vs. ventricle) and session (PRE vs. POST) as a within-subject factors to evaluate the effects of tbTUS in each task.

In the semantic task, there was a significant main effect of session ($F_{1,21} = 10.61$, $p = 0.004$) and an interaction between the stimulation and session ($F_{1,21} = 5.09$, $p = 0.035$) on accuracy. *Post hoc* t-tests revealed that ATL tbTUS significantly increased accuracy in semantic task ($t= -3.66$, $p < 0.001$). No significant effects were found in the control task (Fs < 1.73, ps > 0.202).

For reaction time (RT), we only found a significant main effect of session in both tasks (semantic: $F_{1,21} = 88.56$, $p < 0.001$; control: $F_{1,21} = 8.56$, $p = 0.008$). *Post hoc* t-tests showed that participants responded faster in the post-session, regardless of stimulation type or task (ATL stimulation – semantic: $t = 11.13$, $p < 0.001$; Ventricle stimulation – semantic: $t = 6.39$, $p < 0.001$; ATL stimulation – control: $t = 2.37$, $p = 0.027$; ventricle stimulation – control: $t = 3.05$, $p = 0.006$).

**Supplementary Figure 1**

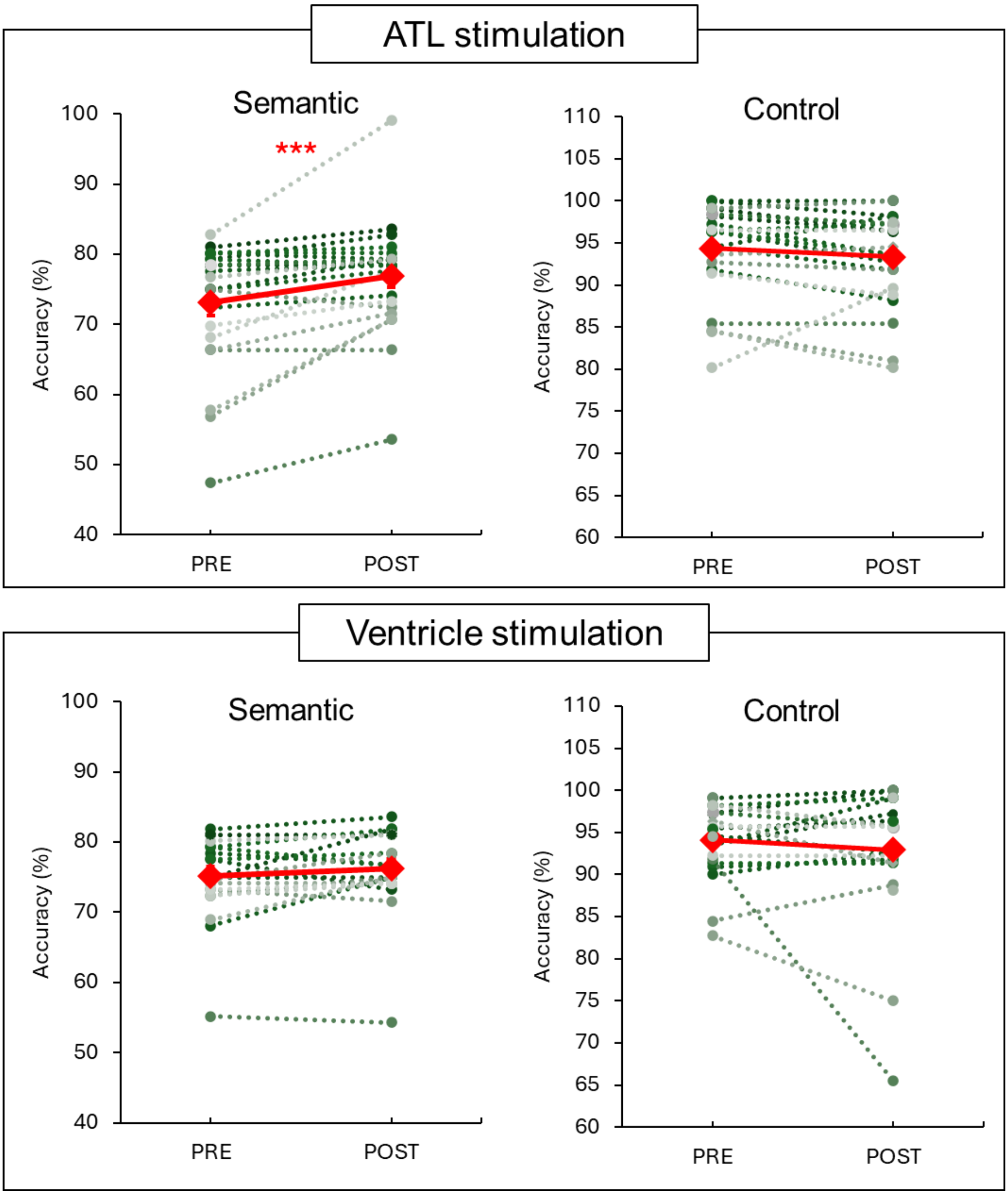


**Figure S1.** Task performance (N = 23): accuracy. ATL stimulation: semantic task, $t(22) = -3.66$, $p < 0.001$; control task, $t(22) = 1.56$, $p = 0.125$. Ventricle stimulation: semantic task, $t(21) = -1.76$, $p = 0.093$; control task, $t(21) = 0.87$, $p = 0.396$. Red diamonds represent the mean of data. Circles represent each individual data. *** $p < 0.001$

**Supplementary Figure 2**

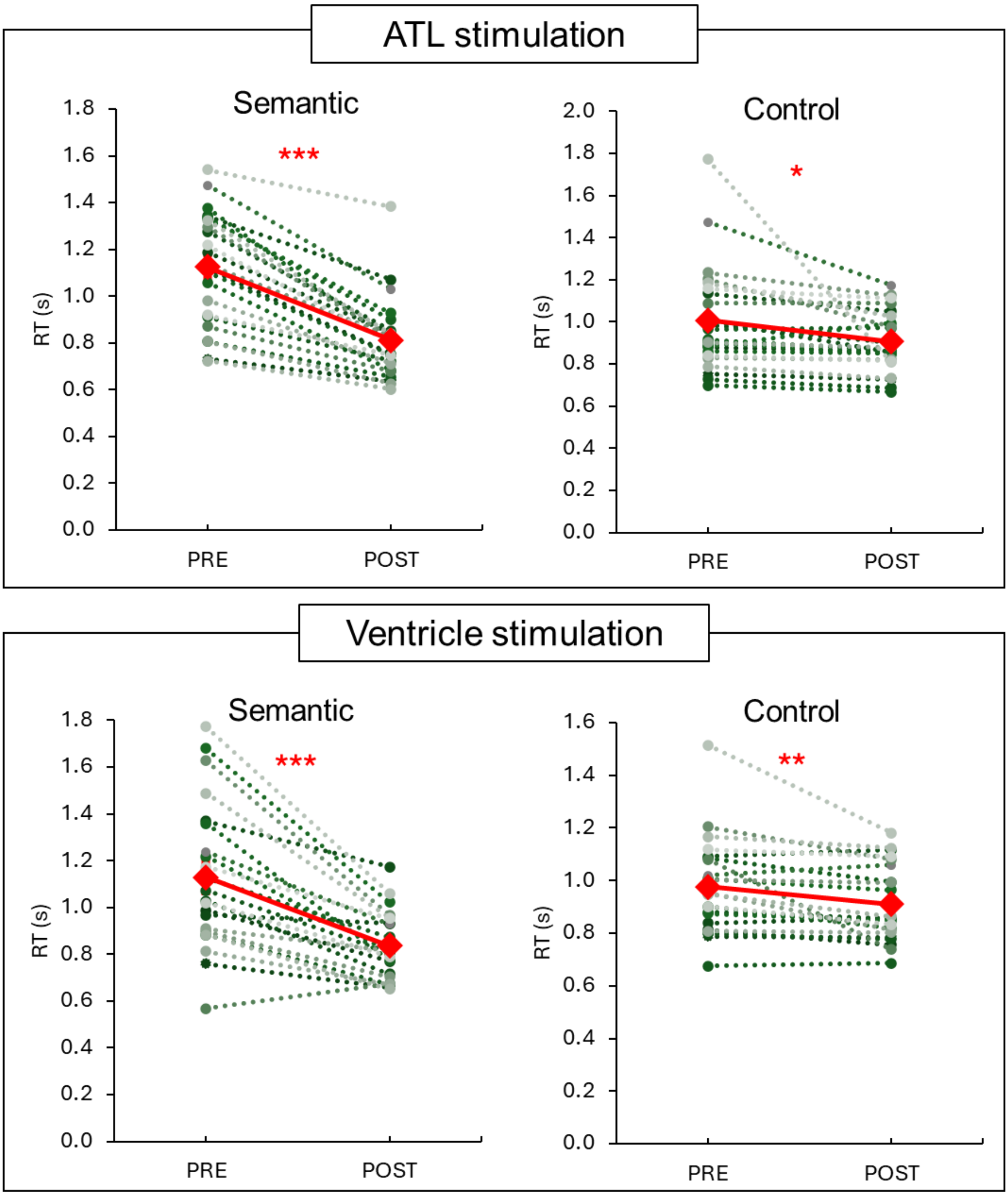


**Figure S2.** Task performance (N = 23): RT. ATL stimulation: semantic task, $t(22) = 11.13$, $p < 0.001$; control task, $t(22) = 2.37$, $p = 0.027$. Ventricle stimulation: semantic task $t(21) = 6.39$, $p < 0.001$; control task: $t(21) = 3.05$, $p = 0.006$. Red diamonds represent the mean of data. Circles represent each individual data. *** $p < 0.001$, ** $p < 0.01$, * $p < 0.05$

**Supplementary Figure 3**

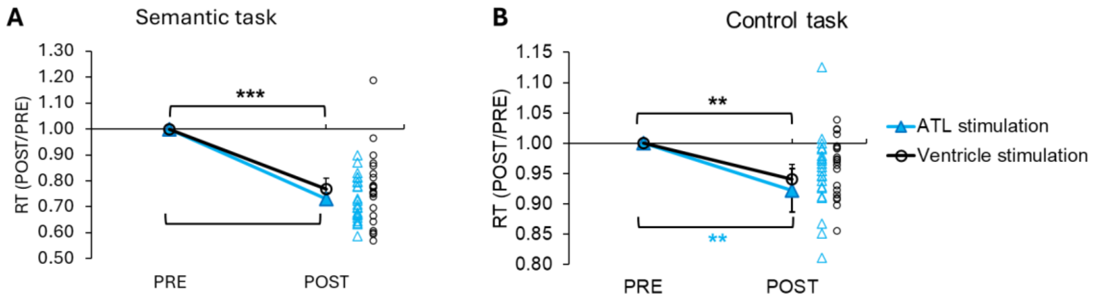


**Figure S3.** A) tbTUS-induced changes in the normalised RT in the semantic task. ATL stimulation: $t(22) = -15.56$, $p < 0.001$; ventricle stimulation: $t(21) = -7.76$, $p < 0.001$. (B) tbTUS-induced changes in the normalised RT in the control (pattern matching) task. ATL stimulation: $t(22) = -3.04$, $p = 0.006$; ventricle stimulation: $t(21) = -3.32$, $p = 0.003$. Light blue lines and triangles indicate the ATL stimulation. Black lines and circles represent the control (ventricle) stimulation. Error bars represent standard error. *** $p < 0.001$, ** $p < 0.01$

**Supplementary Figure 4**

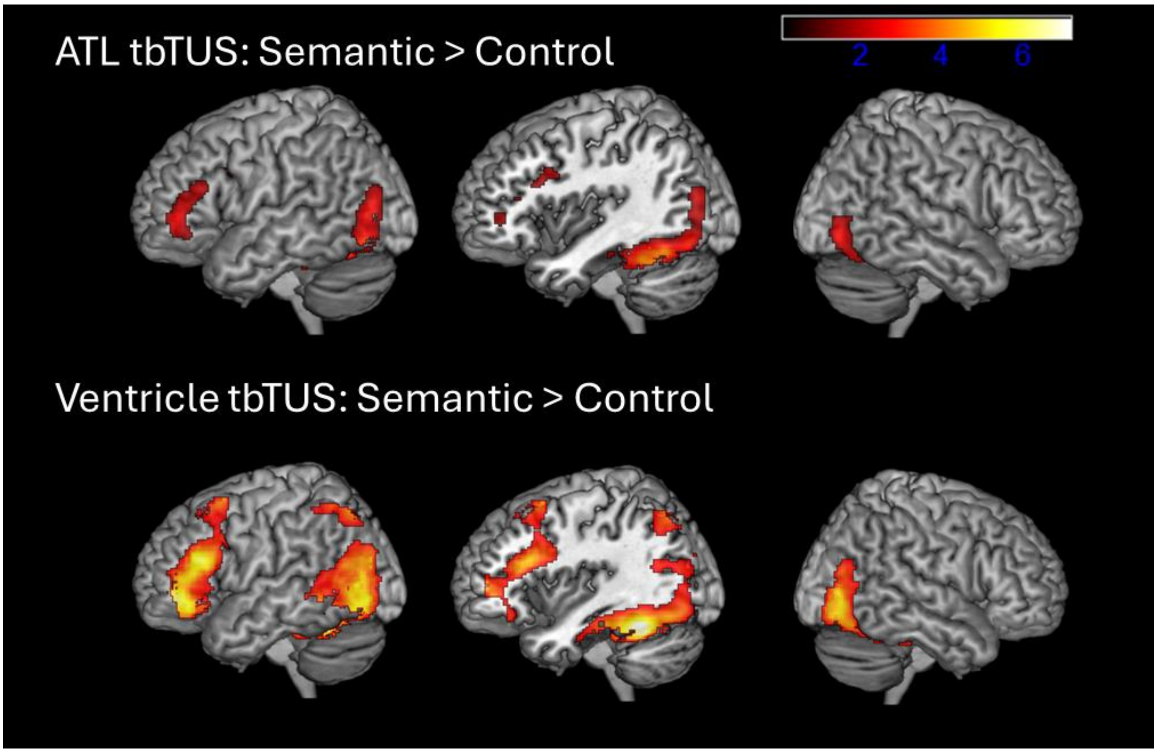


**Figure S4.** The result of fMRI for the contrast of Semantic > Control. The colour bar indicates T score.

**Supplementary Figure 5**

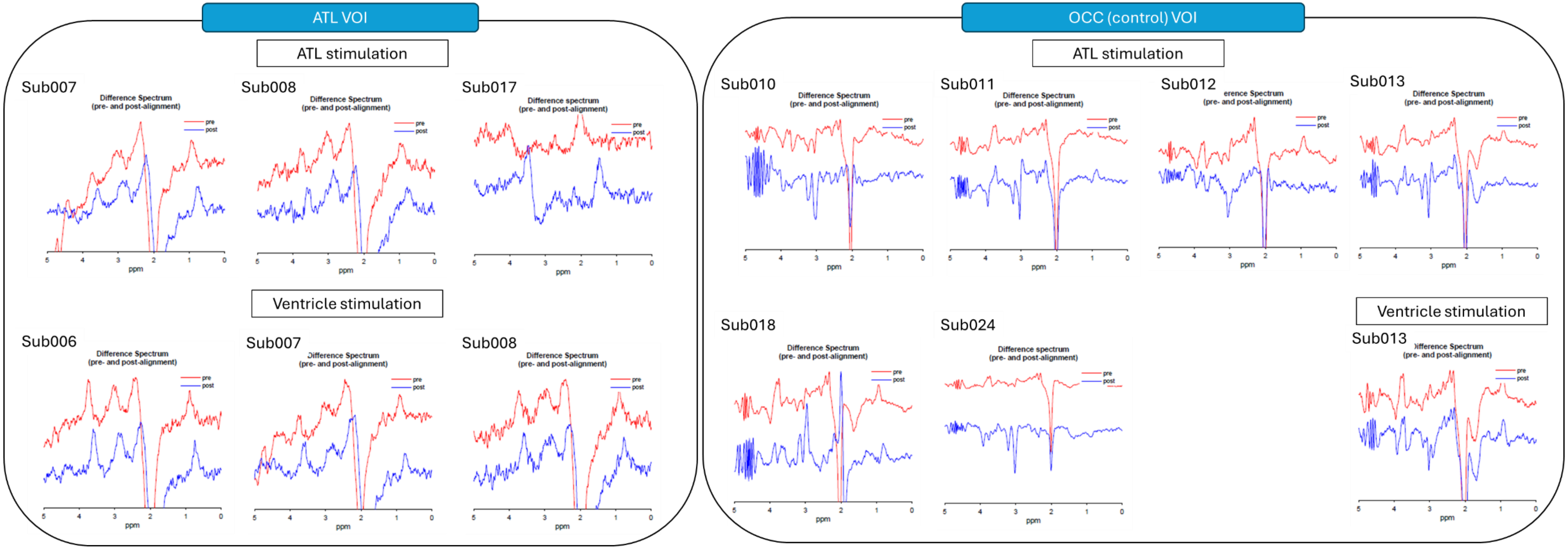


**Figure S5.** The excluded MRS data. The red line represents the raw spectra, and the blue line shows the post-processed spectra.

## Supplementary Table 1

| Region | sonication | N | Data quality metrics | | | | | | | | Tissue segmentations | | |
|---|---|---|---|---|---|---|---|---|---|---|---|---|---|
| | | | Linewidth (FWHM) | SNR | Fit error (%) | | | | | | GM (%) | WM (%) | CSF (%) |
| | | | Water | Water | Water | GABA+ | glx | NAA | tCr | cho | | | |
| ATL | ATL tbTUS | 20 | 23.96 (4.62) | 25314 (8255) | 0.74 (0.35) | 6.67 (1.19) | 5.30 (1.60) | 1.40 (0.50) | 2.31 (0.72) | 4.68 (0.72) | 0.49 (0.04) | 0.49 (0.04) | 0.02 (0.01) |
| | Ventricle tbTUS | 19 | 28.57 (7.79) | 20763 (6143) | 0.78 (0.42) | 8.24 (3.33) | 6.01 (2.66) | 1.35 (0.78) | 2.12 (0.89) | 4.79 (0.87) | 0.49 (0.03) | 0.48 (0.03) | 0.02 (0.02) |
| OCC | ATL tbTUS | 17 | 10.97 (1.03) | 86252 (24501) | 0.45 (0.10) | 2.58 (0.56) | 2.31 (0.44) | 1.01 (0.34) | 1.68 (0.23) | 6.73 (0.72) | 0.69 (0.03) | 0.23 (0.03) | 0.07 (0.03) |
| | Ventricle tbTUS | 21 | 10.93 (1.39) | 80140 (16955) | 0.43 (0.89) | 2.47 (0.54) | 2.09 (0.50) | 1.93 (0.37) | 1.60 (0.19) | 6.76 (0.71) | 0.68 (0.03) | 0.24 (0.03) | 0.07 (0.03) |

Table S1. The summary of MRS data quality metrics and tissue segmentation information. Data are shown as the mean (SD).

## Supplementary Table 2

| Parameter Intrinsic connectivity | ATL stimulation Mean | SD | t | p | Ventricle (control) stimulation Mean | SD | t | p | ATL stimulation vs. Ventricle stimulation t | p |
|---|---|---|---|---|---|---|---|---|---|---|
| L.ATL → R.ATL | 0.088 | 0.028 | 15.284 | <0.001 | 0.015 | 0.003 | 26.663 | <0.001 | 12.490 | <0.001 |
| L.ATL → L.IFG | 0.104 | 0.027 | 18.742 | <0.001 | 0.014 | 0.002 | 32.692 | <0.001 | 16.513 | <0.001 |
| L.ATL → R.IFG | 0.102 | 0.039 | 12.545 | <0.001 | 0.014 | 0.002 | 34.313 | <0.001 | 10.843 | <0.001 |
| L.ATL → L.pMTG | 0.062 | 0.040 | 7.535 | <0.001 | 0.016 | 0.002 | 30.652 | <0.001 | 5.790 | 0.013 |
| L.ATL → R.pMTG | 0.041 | 0.044 | 4.409 | <0.001 | 0.016 | 0.002 | 33.959 | <0.001 | 2.696 | <0.001 |
| R.ATL → L.ATL | 0.122 | 0.040 | 14.642 | <0.001 | 0.015 | 0.003 | 26.771 | <0.001 | 12.570 | <0.001 |
| R.ATL → L.IFG | 0.081 | 0.033 | 11.878 | <0.001 | 0.015 | 0.002 | 39.399 | <0.001 | 10.145 | <0.001 |
| R.ATL → R.IFG | 0.091 | 0.040 | 10.975 | <0.001 | 0.014 | 0.002 | 41.191 | <0.001 | 9.433 | <0.001 |
| R.ATL → L.pMTG | 0.093 | 0.043 | 10.513 | <0.001 | 0.016 | 0.002 | 37.783 | <0.001 | 8.760 | <0.001 |
| R.ATL → R.pMTG | 0.086 | 0.055 | 7.592 | <0.001 | 0.016 | 0.002 | 42.359 | <0.001 | 6.101 | <0.001 |
| L.IFG → L.ATL | 0.132 | 0.034 | 18.674 | <0.001 | 0.015 | 0.002 | 32.672 | <0.001 | 16.678 | <0.001 |
| L.IFG → R.ATL | 0.070 | 0.034 | 10.025 | <0.001 | 0.015 | 0.002 | 39.115 | <0.001 | 8.015 | <0.001 |
| L.IFG → R.IFG | 0.095 | 0.028 | 15.971 | <0.001 | 0.014 | 0.001 | 48.892 | <0.001 | 13.239 | <0.001 |
| L.IFG → L.pMTG | 0.093 | 0.036 | 12.483 | <0.001 | 0.015 | 0.002 | 44.159 | <0.001 | 10.359 | <0.001 |
| L.IFG → R.pMTG | 0.100 | 0.038 | 12.811 | <0.001 | 0.016 | 0.002 | 48.533 | <0.001 | 10.825 | <0.001 |
| R.IFG→ L.ATL | 0.139 | 0.047 | 14.326 | <0.001 | 0.014 | 0.002 | 34.124 | <0.001 | 12.784 | <0.001 |
| R.IFG → R.ATL | 0.083 | 0.029 | 13.799 | <0.001 | 0.014 | 0.002 | 41.184 | <0.001 | 11.123 | <0.001 |
| R.IFG → L.IFG | 0.094 | 0.030 | 15.105 | <0.001 | 0.014 | 0.001 | 48.779 | <0.001 | 12.888 | <0.001 |
| R.IFG → L.pMTG | 0.085 | 0.034 | 11.999 | <0.001 | 0.015 | 0.002 | 48.345 | <0.001 | 9.774 | <0.001 |
| R.IFG → R.pMTG | 0.077 | 0.059 | 6.296 | <0.001 | 0.016 | 0.001 | 50.755 | <0.001 | 4.975 | <0.001 |
| L.pMTG → L.ATL | 0.135 | 0.045 | 14.547 | <0.001 | 0.016 | 0.002 | 31.618 | <0.001 | 12.419 | <0.001 |
| L.pMTG → R.ATL | 0.090 | 0.054 | 7.901 | <0.001 | 0.016 | 0.002 | 39.670 | <0.001 | 6.481 | <0.001 |
| L.pMTG → L.IFG | 0.101 | 0.028 | 16.961 | <0.001 | 0.015 | 0.002 | 45.684 | <0.001 | 14.778 | <0.001 |
| L.pMTG → R.pMTG | 0.083 | 0.048 | 8.256 | <0.001 | 0.015 | 0.001 | 50.875 | <0.001 | 6.706 | <0.001 |
| L.pMTG → R.pMTG | 0.096 | 0.068 | 6.781 | <0.001 | 0.017 | 0.002 | 53.068 | <0.001 | 5.595 | <0.001 |
| R.pMTG→ L.ATL | 0.123 | 0.039 | 15.008 | <0.001 | 0.016 | 0.002 | 34.220 | <0.001 | 12.575 | <0.001 |
| R.pMTG → R.ATL | 0.086 | 0.051 | 8.116 | <0.001 | 0.016 | 0.002 | 41.485 | <0.001 | 6.533 | <0.001 |
| R.pMTG → L.IFG | 0.113 | 0.052 | 10.330 | <0.001 | 0.016 | 0.002 | 48.362 | <0.001 | 8.966 | <0.001 |
| R.pMTG → R.IFG | 0.077 | 0.070 | 5.258 | <0.001 | 0.016 | 0.001 | 51.017 | <0.001 | 4.185 | <0.001 |
| R.pMTG → R.pMTG | 0.103 | 0.075 | 6.610 | <0.001 | 0.017 | 0.002 | 50.808 | <0.001 | 5.547 | <0.001 |

Table S2. Results of intrinsic connectivity. L = left hemisphere, R = right hemisphere

**Supplementary Table 3**

| Semantic task | ATL stimulation | | | | Ventricle (control) stimulation | | | | ATL stimulation vs. Ventricle stimulation | |
|---|---|---|---|---|---|---|---|---|---|---|
| Parameter<br>Modulatory connectivity | Mean | SD | t | p | Mean | SD | t | p | t | p |
| L.ATL → R.ATL | -0.677 | 1.276 | -2.545 | 0.018 | 0.006 | 0.005 | 5.739 | <0.001 | -2.566 | 0.018 |
| L.ATL → L.IFG | -0.488 | 0.657 | -3.561 | 0.002 | 0.004 | 0.002 | 8.395 | <0.001 | -3.588 | 0.002 |
| L.ATL → R.IFG | -0.473 | 0.727 | -3.120 | 0.005 | 0.004 | 0.002 | 9.663 | <0.001 | -3.145 | 0.005 |
| L.ATL → L.pMTG | -1.707 | 0.845 | -9.686 | <0.001 | 0.006 | 0.003 | 10.913 | <0.001 | -9.721 | <0.001 |
| L.ATL → R.pMTG | -2.389 | 0.983 | -11.657 | <0.001 | 0.007 | 0.004 | 7.160 | <0.001 | -11.685 | <0.001 |
| R.ATL → L.ATL | 1.159 | 0.668 | 8.316 | <0.001 | 0.007 | 0.006 | 5.385 | <0.001 | 8.226 | <0.001 |
| R.ATL → L.IFG | -0.134 | 0.236 | -2.715 | 0.013 | 0.004 | 0.002 | 8.436 | <0.001 | -2.805 | 0.01 |
| R.ATL → R.IFG | -0.125 | 0.205 | -2.918 | 0.008 | 0.004 | 0.002 | 10.918 | <0.001 | -3.020 | 0.006 |
| R.ATL → L.pMTG | -0.309 | 0.259 | -5.704 | <0.001 | 0.006 | 0.002 | 12.545 | <0.001 | -5.820 | <0.001 |
| R.ATL → R.pMTG | -0.382 | 0.202 | -9.084 | <0.001 | 0.007 | 0.005 | 6.937 | <0.001 | -9.214 | <0.001 |
| L.IFG → L.ATL | 1.329 | 0.445 | 14.333 | <0.001 | 0.004 | 0.003 | 7.442 | <0.001 | 14.269 | <0.001 |
| L.IFG → R.ATL | -0.220 | 0.202 | -5.221 | <0.001 | 0.004 | 0.002 | 11.088 | <0.001 | -5.314 | <0.001 |
| L.IFG → R.IFG | -0.186 | 0.165 | -5.397 | <0.001 | 0.004 | 0.001 | 12.879 | <0.001 | -5.509 | <0.001 |
| L.IFG → L.pMTG | -0.323 | 0.152 | -10.180 | <0.001 | 0.005 | 0.002 | 14.317 | <0.001 | -10.320 | <0.001 |
| L.IFG → R.pMTG | -0.393 | 0.151 | -12.484 | <0.001 | 0.006 | 0.003 | 8.792 | <0.001 | -12.630 | <0.001 |
| R.IFG→ L.ATL | 1.264 | 0.426 | 14.211 | <0.001 | 0.004 | 0.002 | 8.104 | <0.001 | 14.157 | <0.001 |
| R.IFG → R.ATL | -0.226 | 0.180 | -6.031 | <0.001 | 0.004 | 0.001 | 11.483 | <0.001 | -6.235 | <0.001 |
| R.IFG → L.IFG | -0.207 | 0.094 | -10.544 | <0.001 | 0.004 | 0.001 | 14.800 | <0.001 | -10.708 | <0.001 |
| R.IFG → L.pMTG | -0.304 | 0.138 | -10.525 | <0.001 | 0.005 | 0.002 | 14.629 | <0.001 | -10.666 | <0.001 |
| R.IFG → R.pMTG | -0.337 | 0.172 | -9.376 | <0.001 | 0.006 | 0.003 | 9.526 | <0.001 | -9.498 | <0.001 |
| L.pMTG → L.ATL | 0.836 | 0.347 | 11.557 | <0.001 | 0.006 | 0.004 | 7.523 | <0.001 | 11.428 | <0.001 |
| L.pMTG → R.ATL | -0.074 | 0.270 | -1.312 | 0.203 | 0.006 | 0.002 | 12.679 | <0.001 | -1.414 | 0.171 |
| L.pMTG → L.IFG | 0.014 | 0.178 | 0.387 | 0.702 | 0.006 | 0.002 | 12.837 | <0.001 | 0.229 | 0.821 |
| L.pMTG → R.pMTG | -0.134 | 0.317 | -2.030 | 0.055 | 0.005 | 0.002 | 14.125 | <0.001 | -2.103 | 0.047 |
| L.pMTG → R.pMTG | -0.489 | 0.169 | -13.891 | <0.001 | 0.009 | 0.004 | 10.340 | <0.001 | -14.075 | <0.001 |
| R.pMTG→ L.ATL | 0.534 | 0.281 | 9.099 | <0.001 | 0.008 | 0.002 | 15.766 | <0.001 | 8.940 | <0.001 |
| R.pMTG → R.ATL | -0.082 | 0.325 | -1.206 | 0.24 | 0.008 | 0.003 | 14.160 | <0.001 | -1.313 | 0.203 |
| R.pMTG → L.IFG | 0.133 | 0.331 | 1.919 | 0.068 | 0.007 | 0.002 | 14.101 | <0.001 | 1.818 | 0.083 |
| R.pMTG → R.IFG | -0.179 | 0.412 | -2.089 | 0.048 | 0.007 | 0.002 | 15.356 | <0.001 | -2.163 | 0.042 |
| R.pMTG → R.pMTG | -0.283 | 0.385 | -3.519 | 0.002 | 0.009 | 0.003 | 17.765 | <0.001 | -3.632 | 0.001 |
| Driving input | | | | | | | | | | |
| tbTUS in the L.ATL | -3.589 | 0.240 | -71.784 | <0.001 | | | | | | |

Table S3. Results of modulatory connectivity during semantic processing. L = left hemisphere, R = right hemisphere

**Supplementary Table 4**

| | Ventricle tbTUS | ATL tbTUS | χ2 | p |
|---|---|---|---|---|
| Headache | 0 | 0 | − | − |
| Unusual feeling on the skin | 2 | 3 | 0.178 | 0.673 |
| Neck pain | 2 | 1 | 0.407 | 0.524 |
| Tingling | 3 | 3 | 0.003 | 0.953 |
| Itchiness | 0 | 0 | − | − |
| Difficulty paying attention | 3 | 2 | 0.278 | 0.598 |
| Unusual feelings, attitude, emotions | 0 | 0 | − | − |
| Sleepiness | 5 | 4 | 3.374 | 0.497 |
| Change in hearing | 1 | 0 | 1.069 | 0.301 |
| Nausea/stick to stomach | 0 | 0 | − | − |
| Dizziness | 2 | 0 | 2.188 | 0.139 |
| Anxious/worried/nervous | 0 | 0 | − | − |
| Forgetful | 0 | 0 | − | − |
| Difficulty with your balance | 1 | 1 | 0.001 | 0.974 |
| Other (heard faint clicking/tapping sound during the stimulation) | 17 | 19 | 4.326 | 0.115 |

Table S4. The results of aversive questionnaires. The number was the number of participants.
