# Supplementary material for: Transcranial focused ultrasound stimulation enhances semantic memory by modulating brain morphology, neurochemistry and neural dynamics
Source: Nat Commun. 2026 Feb 16;17:2833. doi: 10.1038/s41467-026-69579-7 (PMC13022233; doi:10.1038/s41467-026-69579-7)
Supplement: Supplementary file 2 — Reporting Summary [file 41467_2026_69579_MOESM2_ESM.pdf]

nature portfolio

Corresponding author(s): Dr JeYoung Jung

Last updated by author(s): Nov 25, 2025

Reporting Summary

Nature Portfolio wishes to improve the reproducibility of the work that we publish. This form provides structure for consistency and transparency in reporting. For further information on Nature Portfolio policies, see our Editorial Policies and the Editorial Policy Checklist.

Statistics

For all statistical analyses, confirm that the following items are present in the figure legend, table legend, main text, or Methods section.

n/a	Confirmed	
☐	☒	The exact sample size (*n*) for each experimental group/condition, given as a discrete number and unit of measurement
☐	☒	A statement on whether measurements were taken from distinct samples or whether the same sample was measured repeatedly
☐	☒	The statistical test(s) used AND whether they are one- or two-sided<br>*Only common tests should be described solely by name; describe more complex techniques in the Methods section.*
☐	☒	A description of all covariates tested
☐	☒	A description of any assumptions or corrections, such as tests of normality and adjustment for multiple comparisons
☐	☒	A full description of the statistical parameters including central tendency (e.g. means) or other basic estimates (e.g. regression coefficient) AND variation (e.g. standard deviation) or associated estimates of uncertainty (e.g. confidence intervals)
☐	☒	For null hypothesis testing, the test statistic (e.g. *F*, *t*, *r*) with confidence intervals, effect sizes, degrees of freedom and *P* value noted<br>*Give P values as exact values whenever suitable.*
☒	☐	For Bayesian analysis, information on the choice of priors and Markov chain Monte Carlo settings
☒	☐	For hierarchical and complex designs, identification of the appropriate level for tests and full reporting of outcomes
☐	☒	Estimates of effect sizes (e.g. Cohen's *d*, Pearson's *r*), indicating how they were calculated

Our web collection on statistics for biologists contains articles on many of the points above.

Software and code

Policy information about availability of computer code

Data collection	Transcranial focused ultrasound was delivered using a four-element CTX-500-4CH transducer coupled with the NeuroFus PRO TPO-203 system (Sonic Concepts, Brainbox Ltd., Cardiff, United Kingdom). Neuroimaging data were acquired using a GE SIGNA Premier 3T MRI scanner equipped with a 48-channel head coil (GE Healthcare, USA). Task presentation and response recording were implemented using PsychoPy (version 2024.2.1).
Data analysis	Acoustic simulations were conducted for all participants using k-Plan v1.2 (Brainbox Ltd., Cardiff, United Kingdom). Voxel-based morphometry (VBM) analyses were performed using CAT12 (https://neuro-jena.github.io/cat12-help/). MRS spectra were analysed using GANNET 3.3.1. fMRI data were pre-processed using the NIHR Nottingham BRC imaging pipeline, as detailed on their GitHub repository (https://github.com/SPMIC-UoN). Statistical analyses were conducted using IBM SPSS Statistics for Windows, version 28 (IBM Corporation, Armonk, NY, USA), R, and SPM12.

For manuscripts utilizing custom algorithms or software that are central to the research but not yet described in published literature, software must be made available to editors and reviewers. We strongly encourage code deposition in a community repository (e.g. GitHub). See the Nature Portfolio guidelines for submitting code & software for further information.

Data

Policy information about availability of data

All manuscripts must include a data availability statement. This statement should provide the following information, where applicable:

- Accession codes, unique identifiers, or web links for publicly available datasets
- A description of any restrictions on data availability
- For clinical datasets or third party data, please ensure that the statement adheres to our policy

The data generated in this study have been deposited in the Open Science Framework database under the CC BY 4.0 license (https://doi.org/10.17605/OSF.IO/FVK7C).

Research involving human participants, their data, or biological material

Policy information about studies with human participants or human data. See also policy information about sex, gender (identity/presentation), and sexual orientation and race, ethnicity and racism.

Reporting on sex and gender	Sex was determined based on participant self-report. The study sample consisted of 23 participants (17 female, 6 male). Information on gender identity was not collected. Because the study used a within-subject experimental design, sex was not included as an analytic factor and no sex-based analyses were performed.
Reporting on race, ethnicity, or other socially relevant groupings	NA
Population characteristics	Twenty-three healthy young adults (6 males, mean age = 21 ± 3.02 years, ranging from 19 to 33) were recruited for this study. All participants were right-handed English speakers 91 with no current diagnosis of neurological or psychiatric disorders and were not taking any medications know to influence brain chemistry during the study.
Recruitment	Participants were recruited through advertisements distributed via the University's email circulation lists. All individuals who expressed interest were screened, and those meeting the inclusion criteria were enrolled. Participants received £30 compensation for each session. Potential sources of bias include self-selection, as individuals motivated to participate in research, particularly those within the university community, may be overrepresented in the sample.
Ethics oversight	All research procedures complied with relevant ethical regulations. Informed written consent was obtained from all participants prior to the study, which was approved by the Ethics Committee of the School of Psychology at the University of Nottingham (F1417).

Note that full information on the approval of the study protocol must also be provided in the manuscript.

Field-specific reporting

Please select the one below that is the best fit for your research. If you are not sure, read the appropriate sections before making your selection.

[x] Life sciences [ ] Behavioural & social sciences [ ] Ecological, evolutionary & environmental sciences

For a reference copy of the document with all sections, see nature.com/documents/nr-reporting-summary-flat.pdf

Life sciences study design

All studies must disclose on these points even when the disclosure is negative.

Sample size	Our sample size was determined a priori using data and effect sizes from TMS studies with comparable designs and outcome measures (Jung & Lambon Ralph., 2016; 2021; Jung et al., 2022; 2025).<br>In order to provide robust power calculations for each method we have: (a) utilised data from previous whole experiments; (b) selected experiments which have the same design; (c) for the fMRI analysis, we picked a critical ROI from within the semantic network with the weakest intrinsic fMRI signal (ATL), to be maximally conservative.<br>fMRI: power analysis was calculated based on Mumford & Nichols's fMRI power toolbox (http://fmripower.org/).<br>Semantic vs. control task from the fMRI-TMS data block-fMRI design with N=23 participants for α=0.05, power=80%, interaction TMS (ATL vs OCC) x task effect size = 0.33 in behaviour and fMRI signal, then N > 16 are required.<br>TMS, TMS/fMRI and TMS/fMRI/MRS: The study used a 2 (ATL target vs. control site) × 2 (semantic vs. control task) x session (PRE vs POST) within subject design. The data from N from 21 to 23 participants indicate that to achieve α=0.05, power=80% for the critical interaction (effect size ≈ 0.35) in MRS, behavioural and fMRI measurement then N >18 are required.
Data exclusions	MRS data exclusion criteria included fit errors > 15% for each neurochemical, water linewidths (FWHM) > 20 Hz, and SNR < 40.
Replication	We did not replicate any experiments in individuals.
Randomization	The order of stimulation was counterbalanced across participants

Blinding	The experiment was designed as a single-blind study. Participants were informed that stimulation would be applied to different areas of the semantic network, although experimenters were aware of the stimulation site to ensure accurate targeting. To check blinding integrity, participants were verbally asked at the end of all sessions after being informed that one session involved control stimulation, whether they could distinguish between the two stimulation conditions. No participants were able to identify the control session.

Reporting for specific materials, systems and methods

We require information from authors about some types of materials, experimental systems and methods used in many studies. Here, indicate whether each material, system or method listed is relevant to your study. If you are not sure if a list item applies to your research, read the appropriate section before selecting a response.

Materials & experimental systems

n/a	Involved in the study
☒	☐ Antibodies
☒	☐ Eukaryotic cell lines
☒	☐ Palaeontology and archaeology
☒	☐ Animals and other organisms
☒	☐ Clinical data
☒	☐ Dual use research of concern
☒	☐ Plants

Methods

n/a	Involved in the study
☒	☐ ChIP-seq
☒	☐ Flow cytometry
☐	☒ MRI-based neuroimaging

Plants

Seed stocks	*Report on the source of all seed stocks or other plant material used. If applicable, state the seed stock centre and catalogue number. If plant specimens were collected from the field, describe the collection location, date and sampling procedures.*
Novel plant genotypes	*Describe the methods by which all novel plant genotypes were produced. This includes those generated by transgenic approaches, gene editing, chemical/radiation-based mutagenesis and hybridization. For transgenic lines, describe the transformation method, the number of independent lines analyzed and the generation upon which experiments were performed. For gene-edited lines, describe the editor used, the endogenous sequence targeted for editing, the targeting guide RNA sequence (if applicable) and how the editor was applied.*
Authentication	*Describe any authentication procedures for each seed stock used or novel genotype generated. Describe any experiments used to assess the effect of a mutation and, where applicable, how potential secondary effects (e.g. second site T-DNA insertions, mosiacism, off-target gene editing) were examined.*

Magnetic resonance imaging

Experimental design

Design type	Within-subject block design task-fMRI
Design specifications	. For fMRI, participants completed the semantic and control tasks in a block design. Each task block consisted of four trials, and eleven blocks of each task were alternated (e.g., A-B-A-B) with a 4000ms fixation period between blocks. Each trial began with a 500 ms fixation period, followed by a 3500 ms presentation of the stimuli. Each task had 44 trials, and the total fMRI task took about 7 minutes 30s.
Behavioral performance measures	For behavioural data, accuracy and reaction time (RT) were calculated for each individual. A 2 × 2 repeated measures ANOVA with stimulation (ATL vs. ventricle) and session (PRE vs. POST) as within-subject factors was performed on each task (semantic and control). Planned paired t-tests were conducted to examine tbTUS effects in pre- and post-stimulation sessions.

Acquisition

Imaging type(s)	Structural, functional, and single-voxel spectroscopy
Field strength	3T
Sequence & imaging parameters	Images were acquired using a General Electric (GE) SIGNA Premier 3T MR scanner with a 48-channel head coil (GE Healthcare, USA). T1-weighted images were obtained using 3D MPRAGE sequence (voxel size = 1mm isotropic, field of view [FOV] = 256, matrix = 256, 256 sagittal slices, inverse time [TI] = 800ms, flip angle [FA] = 8°).<br>MRS data were collected using a GABA-edited MEGA-PRESS sequence (TR = 2000ms, TE = 68ms) 98. The voxel of interest (VOI) was manually positioned in the left ventrolateral ATL (voxel size = 40 x 20 x 20mm), avoiding overlap with the hippocampus and occipital cortex (OCC) (voxel size = 30 x 30 x 30mm) (Fig. 1A). Spectra were acquired in interleaved blocks of four scans with the MEGA inversion pulses applied at 1.95 ppm to edit GABA signal. This included 184 repeats at the ATL VOI and 112 repeats at the OCC VOI. The protocol provided robust measurements of GABA and glx concentrations from the ATL VOI 26, 99, 100. A total of 4096 sample points were collected at a spectral width of 5 kHz.

fMRI data were collected using a whole- brain 2D GE-EPI sequence (TR = 1400ms, TE = 35ms, flip angle = 68°, in-plane FOV = 212 × 212mm, 57 slices, slice thickness = 2 mm, voxel size = 2mm isotropic, hyperband factor = 3, ARC factor =2, 344 volumes, 8.2 mins total scan time). To account for echo-planar imaging (EPI) distortions, two SE-EPI images with opposite phase encoding directions will be acquired, sharing the same geometry, echo spacing, and phase encoding direction parameters as the GE fMRI scans.

Area of acquisition	A whole brain scan was used for structural and functional MRI acquisitions. MR spectroscopy was acquired in two predefined regions of interest, left ventrolateral ATL (voxel size = 40 x 20 x 20mm), avoiding overlap with the hippocampus and occipital cortex (OCC) (voxel size = 30 x 30 x 30mm).

Diffusion MRI ☐ Used ☒ Not used

Preprocessing

Preprocessing software	Voxel-based morphometry (VBM) was utilized to investigate changes in grey matter (GM) and white matter (WM) using CAT12 (https://neuro-jena.github.io/cat12-help/). MRS spectra were analysed using GANNET 3.3.1. fMRI data were pre-processed using the latest version of the NIHR Nottingham BRC imaging pipeline, as detailed on their GitHub page (https://github.com/SPMIC-UoN).
Normalization	Structural and functional data were normalized to the MNI space via CAT12 or NHIR Nottingham BRC imaging pipeline.
Normalization template	MNI152
Noise and artifact removal	Motion outliers were identified and were included as regressors in the model.
Volume censoring	NA

Statistical modeling & inference

Model type and settings	A contrast of interest (sematic > control) for each participant was calculated. One-sample t-test was performed to estimate the contrast of interest at the group-level.
Effect(s) tested	The effects tested was a measure of task-induced activity and effective connectivity between regions of interests.

Specify type of analysis: ☐ Whole brain ☐ ROI-based ☒ Both

Anatomical location(s): Peak coordinates were based on prior studies using the same tasks: ATL (MNI left: -36, -15, -30; right: 33, -6, -36), IFG (MNI left: -48, 21, 24; right: 57, 24, 21), and pMTG (MNI left: -57, -48, -3; right: 54, -69, 12).

Statistic type for inference<br>(See Eklund et al. 2016)	A standard voxel-wise analysis with cluster-level inference was performed, applying a voxel-wise threshold of p < 0.001, corresponding to T > 5.
Correction	Clusters were considered significant when passing a threshold of p FWE-corrected < 0.05, with at least 100 contiguous voxels.

Models & analysis

n/a	Involved in the study
☐	☒ Functional and/or effective connectivity
☒	☐ Graph analysis
☒	☐ Multivariate modeling or predictive analysis

Functional and/or effective connectivity	Effective connectivity: blood oxygenation level-dependent (BOLD) time-series were extracted and converted to neural activity using the first eigenvector in SPM12.
